# A Clinical, Etiological, and Therapeutic Profile of Gynecomastia

**DOI:** 10.7759/cureus.27687

**Published:** 2022-08-04

**Authors:** Lamiaa Elazizi, Mohammed Amine Essafi, Aabi Hanane, Hayat Aynaou, Houda Salhi, Hanan El Ouahabi

**Affiliations:** 1 Department of Endocrinology, Diabetology, Metabolic Diseases and Nutrition, Hassan II University Hospital Center, Fez, MAR

**Keywords:** hormonal therapy, mammography, persistent pubert, hypogonadism, gynecomastia

## Abstract

Background and objective

Gynecomastia is defined as a benign proliferation of male breast glandular tissue, either unilateral or bilateral, resulting from an imbalance of testosterone and estrogen. In this study, we aimed to describe the clinical, etiological, and therapeutic aspects of gynecomastia.

Materials and methods

A retrospective, descriptive study was conducted in the Department of Endocrinology, Diabetology, and Nutrition at the Hassan II University Hospital in Fez, Morocco, over a period of 10 years. We included all patients admitted for exploration and treatment of gynecomastia. The data were analyzed using Microsoft Excel 2016 and SPSS Statistics version 18 (IBM, Armonk, NY).

Results

A total of 86 patients were included in this study; the mean age of the patients was 33 years (range: 15-86 years). A family history of gynecomastia was found in 4.6%. Isolated gynecomastia was the most frequent symptom (60.4% of cases). It was bilateral in 54% of cases, stage II in 63% of patients, stage I in 17%, and stage III in 20%. The first-line assessment (renal insufficiency, hepatic insufficiency/cirrhosis, dysthyroidism)* *was normal in the majority of cases. The etiologies were dominated by hypogonadism in 32.6% of cases, pubertal gynecomastia in 21%, and senile gynecomastia in 8.1%. Regarding treatment, 42% of patients received an etiological treatment, while surgical treatment was provided in 28% of cases, observation in 15% of cases, and androgen therapy in 15%. Pathological examination of all surgical specimens was benign. The follow-up was marked by 30.3% of static gynecomastia, 29% of regression, 17.5% of good response after surgery, and 24.4% of treatment refusal.

Conclusions

It is important to adopt a step-by-step approach in treating gynecomastia, starting with detailed questioning and clinical examination. The surgical treatment is currently the treatment of choice, the final goal of which is good aesthetic as well as psychological outcomes.

## Introduction

Gynecomastia is defined as benign proliferation of male breast glandular tissue, secondary to an imbalance of testosterone and estrogen. The diagnosis is primarily clinical. It is considered physiological at different stages of life. However, it can be pathological or idiopathic. Therefore, it requires a clinical and biochemical evaluation. The treatment is ideally etiological; if necessary, the treatment is symptomatic, most often surgical. The purpose of our study is to describe the clinical, etiological, and therapeutic aspects of gynecomastia.

## Materials and methods

Study design and setting

A retrospective, descriptive study was conducted in the Department of Endocrinology, Diabetology, and Nutrition at the Hassan II University Hospital in Fez, Morocco, over a period of 10 years. We included all patients who presented for an evaluation of gynecomastia. We excluded patients with incomplete records that were not easily usable or patients lost to follow-up.

Data collection and assessment

The data were collected from the medical records and were entered into and analyzed using Microsft Excel.

Sociodemographic Variables

The sociodemographic variables analyzed were age, drugs used, smoking status, and family history.

Clinical Variables

We examined the following clinical variables: gynecomastia, unilateral or bilateral; stage based on the Simon staging; and examination of external genital organs.

Biological Assessment

First-line assessment: total testosterone, gonadotropins [follicle-stimulating hormone (FSH), luteinizing hormone (LH)], estradiol (E2), prolactin (PRL), thyroid-stimulating-hormone (TSH), kidney function and liver function, as well as tumor markers: alpha-fetoprotein (AFP) and human chorionic gonadotropin (hCG).

Morphological Assessment

Breast ultrasound and/or mammography were requested in case of diagnostic doubt. Testicular ultrasound was performed systematically in all patients as part of the etiological assessment of gynecomastia. Hypothalamic-pituitary MRI was performed only in cases with hypogonadotropic hypogonadism.

Diagnosis Retained

The analysis of the clinical and biological data made it possible to retain the diagnosis of physiological gynecomastia, idiopathic or secondary to an endocrine or systemic pathology.

Processing

The patients were classified according to the following therapeutic modalities: etiological treatment, abstention with monitoring, or symptomatic treatment in its two aspects: medical and/or surgical.

Anatomopathological Examination

All excisional specimens were sent for anatomopathological examination.

Statistical analysis and patient consent

The data were analyzed using Excel 2016 and SPSS Statistics version 18 (IBM, Armonk, NY). All patients included in this study gave their oral consent.

## Results

The study included a total of 86 patients, aged 15 to 86 years. The mean age was 33 years. The most represented age group was 15-25 years at a rate of 52%; 26% of the patients were in the age group of 26-49 years and 22% were aged >50 years. The incidence of drug intake was found in 11.6%, while 4.6% had a family history of breast cancer; toxin intake was found in 7%.

Gynecomastia was diagnosed after a progression of three years on average in the cohort. The majority of the patients (98.8%) had progressive disease. Isolated gynecomastia was the most common symptom in our series (60.4% of cases). However, 25.6% of patients reported libido disorders; mastodynia was found in 12.8% of cases and galactorrhea was found in a single patient.

**Figure 1:**
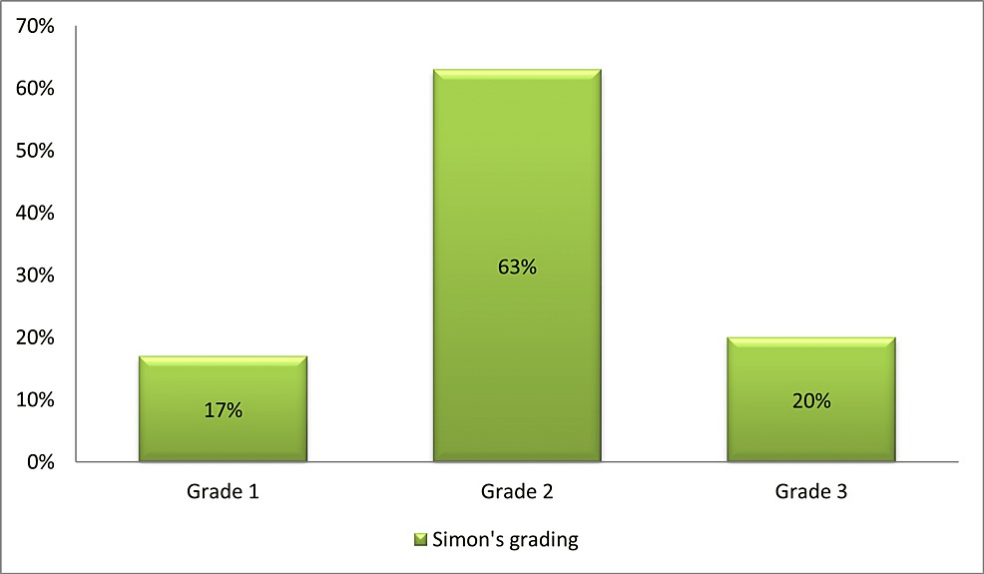
Distribution of patients according to the Simon staging of gynecomastia

**Figure 2:**
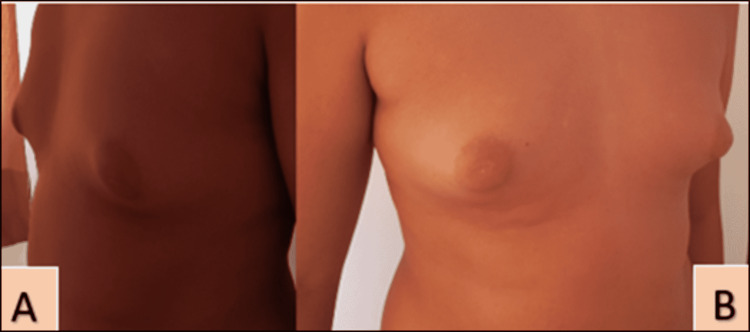
Bilateral gynecomastia stage 2-3 Profile (A) and face (B); Hassan II University Hospital of Fez

On physical examination, gynecomastia was bilateral in 54% of cases; the staging of gynecomastia was based on the Simon stages, as shown in Figures 1-2.

Regarding pubertal development, Tanner's stage 5 was the most represented with a percentage of 78%. The examination of external genital organs demonstrated an anomaly in 22% of the cases, of which micropenis was the most frequently observed one.

**Figure 3:**
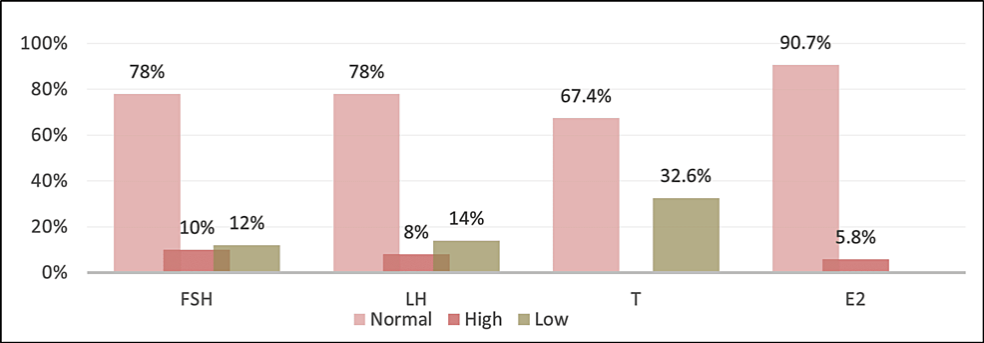
Distribution of patients according to the results of the hormonal assessment FSH: follicle-stimulating hormone; LH: luteinizing hormone; T: testosterone; E2: estradiol

The first-line assessment was normal in the majority of cases. Hypogonadism was found in 32.6% of cases, and tumor markers were negative in 97% of cases (Figure 3).

The distribution of the radiological forms of gynecomastia was as follows: 78% dendritic, 17.4% diffuse, and 4.6% nodular. No signs of microcalcifications or suspicious lesions were found in any of the cases. Testicular ultrasound was performed on all patients. Of note, 40.7% had an anomaly on the testicular ultrasound.

A hypothalamic-pituitary MRI was performed on 23 patients who showed secondary hypogonadism on the hormonal balance sheet; it was abnormal in only 10.5% of cases. The most common anomaly was pituitary adenoma.

**Figure 4:**
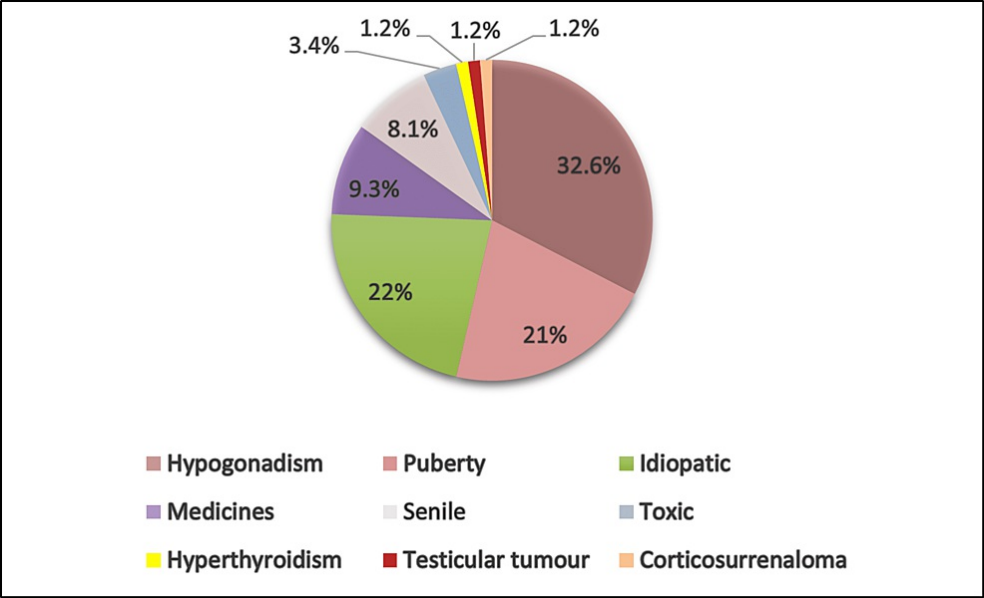
Distribution of patients according to the etiological diagnosis of gynecomastia

Regarding the etiologies, hypogonadism was found in 32.6%, while the etiology was idiopathic in 22% of cases; pubertal gynecomastia was seen in 21%, and senile gynecomastia in 8.1% of cases. The etiology was of toxin origin in 3.4% of cases, drug-induced in 9.3%, and 1.2% of cases had a testicular tumor. An adrenocortical tumor was found in 1.2% of cases, and hyperthyroidism in 1.2% (Figure 4).

For therapeutic management, etiological treatment was employed in 57% of cases while 28% of patients received surgical treatment; follow-up was performed in 15% of cases.

Histological examination of all excision specimens was in favor of benignity. The follow-up was marked by 30.3% stationary gynecomastia, 29% regression, 17.5% good evolution after surgery, and 23.2% of treatment refusal.

## Discussion

Gynecomastia refers to a benign proliferation of breast tissue in men, resulting from an imbalance of the ratio between testosterone and estrogen at the expense of the latter [1].

The prevalence of gynecomastia is estimated to be between 32 and 65%, based on different methods of evaluation and analysis of men of different ages and with different lifestyles [1]. In our study, the average age was 33 years. This finding is similar to that of Costanzo et al. and Mieritz et al. Most of our patients were teenagers, and the most represented age group was 15-25 years with 52% of cases. This corresponds to a case series in the literature that estimated that the frequency of gynecomastia is high at puberty (50-70%) in adolescents with a peak incidence at the age of 13-14 years [4].

Gynecomastia is often discovered during a routine physical exam and is usually asymptomatic. However, some patients may present with complaints of discomfort as it can be a source of psychological problems. In our series, the most frequent symptom was isolated gynecomastia (60.4% of cases). This could be explained by the fact that it is an aesthetic prejudice. This aligns with the study by Costanzo et al. who found that the top complaints in their series involved cosmetic issues [2]. In our series, the average time to consultation after the onset was three years (47% of cases). 

The literature reports that the condition is bilateral in about half of the patients [5]; in our series, gynecomastia was bilateral in 54% of cases. However, Mannu et al. reported a clear predominance of the unilateral nature of gynecomastia in their study (71.6% of cases) [6].

There are several morphological classifications of gynecomastia involved in the choice of the appropriate surgical technique; in our series, the majority of patients had stage II per the Simon grading (63% of cases), which is consistent with the findings of Barros et al., Choi et al., and Li et al, who found stage II disease in 49.3%, 68%, and 70% of cases respectively [5,7,8].

In our series, gynecomastia was idiopathic in 22% of cases and pubertal in 21%. In 48.8% of cases, the etiology was distributed as follows: hypogonadism in 32.6% of cases including 26.8% secondary and 5.8% primary with four cases of Klinefelter syndrome; drug-induced in 9.3%, senile in 8.1%, of toxin origin in 3.4%, hyperthyroidism in 1.2%, testicular tumor in 1.2%, and adrenocortical tumor in 1.2% of cases. This is consistent with the data in the literature, which report that idiopathic gynecomastia has a prevalence of 25-30%, persistent pubertal gynecomastia accounts for 15-25% of cases, while there are 3% with tumor causes and 1.5% with hyperthyroidism [9].

However, some other studies in the literature report a prevalence of hypogonadotropic hypogonadism of 8%, hypergonadotropic of 2%, and of drug-induced gynecomastia of more than 20%. In 15%, gynecomastia is caused by other causes as diverse as chronic renal failure (1%), hepatic failure (1%), androgen insensitivity syndrome, true hermaphroditism, and aromatase hyperactivity [4,6,10,11].

Management of gynecomastia

Etiological treatment was employed in 36 patients (42% of cases). Therapeutic abstention with monitoring was done in 13 patients (15% of cases), while medical treatment based on dihydrotestosterone was chosen in 13 patients (15% of cases) and surgery was oped for in 24 patients (28% of cases).

These results are consistent with those of the study by Bchir et al. [12], involving 35 cases of gynecomastia, where 17% of patients benefited from surgical treatment, 20% from medical treatment based on local androgen, while abstention with monitoring was done in 52% of cases and discontinuation of drug was involved in 11% of cases (Table 1).

**Table 1:** Treatment of gynecomastia in various studies

Study	Medical treatment	Surgical treatment	Monitoring	Etiological treatment
Messaoudi et al. [13]	33.3%	33.3%	-	33.3%
Bchir et al. [12]	20%	17%	52%	11%
Malhotra et al. [14]	5%	44%	51%	-
Our series	15%	28%	15%	42%

In our series, the histopathological examination of all excision specimens was in favor of benign gynecomastia without any evidence of malignancy. This agrees with the findings of Lapid and Jolink, Boljanovic et al., Choi et al., and Innocenti et al., whereas Handschin et al. diagnosed atypical cell lesions in 3% of patients in their series [15,16,17,18].

Study limitations

This study was limited by the small sample size, which could be attributed to the low number of consultations related to this condition as many men are uncomfortable when it comes to seeking medical help for gynecomastia.

## Conclusions

Based on our findings, the evaluation of gynecomastia can be complex. As it can reveal an underlying pathology or accompany a life-threatening condition, it is critical to timely diagnose and treat gynecomastia. A step-by-step approach toward treatment starting with detailed questioning and clinical examination can avoid the need for an in-depth check-up. The appropriate medical treatment for this condition is still under debate because of its late and ambiguous results. Surgical treatment is currently the treatment of choice, the final goal of which is favorable aesthetic as well as psychological outcomes.
